# Long-term outcome of combined orthograde and surgical management of perforating internal root resorption using a bioceramic repair material: an eight-year CBCT-documented case report

**DOI:** 10.1186/s12903-026-08452-x

**Published:** 2026-04-29

**Authors:** Mohammed Howait, Loai Alsofi

**Affiliations:** Department of Endodontics, Faculty of Dentistry, King Abdulaziz University, P.O. Box 80209, Jeddah, 21589 Saudi Arabia

**Keywords:** Internal root resorption, CBCT, Bioceramic materials, EndoSequence Root Repair Material, Endodontic microsurgery, Long-term follow-up.

## Abstract

**Background:**

Perforating internal root resorption (IRR) represents a rare and challenging endodontic condition that may require a combined nonsurgical and surgical approach for effective management.

**Methods:**

A 24-year-old female presented with pain on biting and discoloration of the maxillary right lateral incisor. Cone-beam computed tomography (CBCT) confirmed perforating IRR associated with apical periodontitis. Initial non-surgical disinfection was performed using calcium hydroxide as an intracanal medicament. Persistent intracanal exudation necessitated surgical intervention under an operating microscope, during which the defect was sealed with EndoSequence Root Repair Material (ERRM) Putty and covered with a bone graft. Final obturation was completed using a hydraulic condensation technique with a bioceramic sealer.

**Results:**

At the eight-year follow-up, the tooth remained functional and asymptomatic. Both periapical radiographs and CBCT imaging demonstrated complete osseous healing and restoration of normal trabecular bone pattern.

**Conclusion:**

This case illustrates that perforating IRR may be successfully managed using a combined non-surgical and surgical approach guided by CBCT and supported by bioactive bioceramic materials, with stable clinical and radiographic outcomes documented over an eight-year follow-up period.

## Introduction

Internal root resorption (IRR) is a rare but progressive form of dental hard tissue loss that originates from the pulp space and is mediated by odontoclastic activity secondary to chronic inflammation or trauma [1–5]. In the early stages, IRR is often asymptomatic and may be detected incidentally on routine radiographic examination; however, once the resorptive process progresses and perforates the root surface, the clinical management and prognosis become considerably more complex [2, 6].

Several etiological factors have been associated with the development of IRR, including dental trauma [7], chronic pulpal inflammation [8], and orthodontic tooth movement [9]. In the present case, the patient reported a previous history of orthodontic treatment, which has been identified as a potential contributing factor for root resorption. Orthodontic tooth movement can induce inflammatory responses in the periodontal ligament and dental pulp due to mechanical stress and vascular alterations [10]. These biological responses may stimulate clastic cell activity, leading primarily to external root resorption; however, IRR has also been reported in association with orthodontic therapy, particularly when additional predisposing factors such as dental trauma, pulpal irritation, or restorative procedures are present [11–13]. Therefore, a careful evaluation of patient history, including prior orthodontic treatment, is important when assessing resorptive defects.

Conventional two-dimensional radiographs often fail to accurately determine the extent and perforation status of internal resorptive lesions. They typically reveal a round or oval radiolucent enlargement of the pulp space but cannot reliably distinguish between internal and external resorption or accurately demonstrate the extent of the defect and its relationship to surrounding hard tissues [14, 15]. Accurate diagnosis is critical to prevent unnecessary tooth loss [2, 4], and cone-beam computed tomorgraphy (CBCT) has become an important diagnostic adjunct in cases where perforation is suspected, as it directly influences both the treatment approach and the decision to incorporate surgical intervention [15–18].

Management of perforating IRR requires effective disinfection of the root canal system combined with a hermetic seal of the resorptive defect. Traditionally, mineral trioxide aggregate (MTA) has been the material of choice because of its proven biocompatibility and sealing ability [19, 20]. Long-term follow-up reports of perforating IRR managed using contemporary calcium silicate-based bioceramic materials remain relatively limited in the literature. Most available case reports describe short-term outcomes or focus primarily on the technical management of the defect rather than long-term clinical performance of the treated tooth. Recent case reports have demonstrated that perforating IRR may be successfully managed using conservative nonsurgical approaches with bioactive materials when accurate diagnosis and appropriate disinfection protocols are achieved [21, 22].

More recently, calcium silicate-based bioceramic repair materials, such as EndoSequence Root Repair Material (ERRM), have demonstrated favorable handling characteristics, bioactivity, and long-term stability, making them suitable alternatives to MTA [23–25].

Although several case reports have described the management of internal root resorption, reports documenting long-term outcomes following combined orthograde and surgical management of perforating internal root resorption remain limited, particularly when modern calcium silicate-based bioceramic repair materials are used. Long-term follow-up data are important to better understand the durability of such treatment approaches.

The present case report describes the diagnosis and management of perforating internal root resorption in a maxillary incisor using a combined nonsurgical and surgical approach. The perforation was repaired using a bioactive calcium silicate-based bioceramic material, and the clinical and radiographic outcomes were evaluated over an eight-year follow-up period. This report highlights the diagnostic value of CBCT and demonstrates the potential for favorable long-term outcomes when perforating IRR is managed using a combined approach supported by modern bioactive repair materials.

## Case report

### Patient and diagnosis

A 24-year-old medically healthy female patient presented with mild discomfort during biting and discoloration of the maxillary right lateral incisor (tooth #7). The tooth had previously received a Class III composite restoration. The patient also reported a history of previous orthodontic treatment, which has been suggested as a potential predisposing factor for root resorption in susceptible teeth. The patient’s medical history was non-contributory. The patient’s dental history revealed previous orthodontic treatment and restorative procedures.

Extraoral clinical examination was normal. Intraoral clinical examination revealed tenderness to percussion without swelling or the presence of a sinus tract. The tooth was non-responsive to pulp sensibility testing, whereas adjacent teeth responded within normal limits. Periodontal probing depths were within normal physiological limits. Based on the clinical findings, a diagnosis of pulp necrosis associated with symptomatic apical periodontitis was established.

### Radiographic and CBCT evaluation

**Fig. 1:**
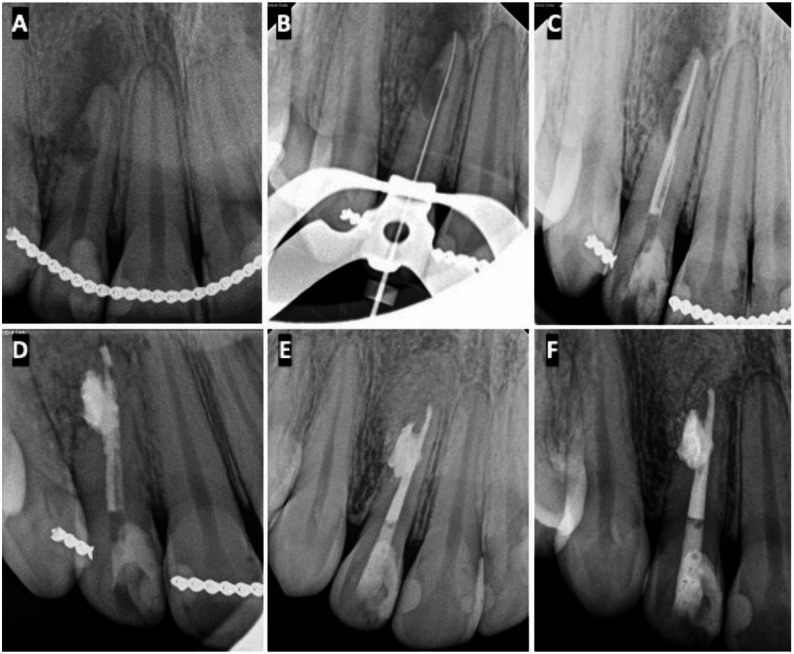
Radiographic sequence demonstrating the diagnosis, treatment procedures, and long-term outcome of perforating internal root resorption in a maxillary lateral incisor (tooth #7). **A** Preoperative periapical radiograph showing a well-defined radiolucent enlargement of the root canal space in the middle third of the root, consistent with internal root resorption and suggestive of possible perforation of the root wall. A periapical radiolucency associated with the same tooth is also evident. **B** Working length determination during endodontic treatment using an intracanal file, confirming canal negotiation and preparation. **C** Placement of calcium hydroxide as an intracanal medicament. **D** Post-surgical periapical radiograph demonstrating repair of the perforating resorptive defect using a calcium silicate-based bioceramic material following surgical access, debridement, and bone graft placement. Root-end cavity preparation and retrograde filling were also performed under magnification during the surgical procedure. **E** Immediate postoperative radiograph after completion of root canal obturation and coronal restoration, confirming adequate sealing of the canal system and repair site. **F** Eight-year follow-up radiograph demonstrating complete resolution of the periapical radiolucency and stable repair of the resorptive defect, indicating long-term clinical and radiographic stability

A preoperative periapical radiograph revealed a well-defined radiolucent enlargement in the middle third of the root extending toward the apical region, suggestive of a resorptive defect (Fig. 1A). However, the two-dimensional radiograph could not reliably determine the full extent of the lesion or confirm possible perforation of the root surface.

To obtain a three-dimensional assessment of the defect, cone-beam computed tomography (CBCT) imaging was performed using an iCAT CBCT scanner (Imaging Sciences International, Hatfield, PA, USA) with a voxel size of 0.2 mm and a limited field of view (5 × 5 cm). The limited field of view was selected to minimize radiation exposure while providing adequate diagnostic information. The CBCT examination was justified based on diagnostic necessity and performed in accordance with the ALARA (As Low As Reasonably Achievable) principle.

**Fig. 2:**
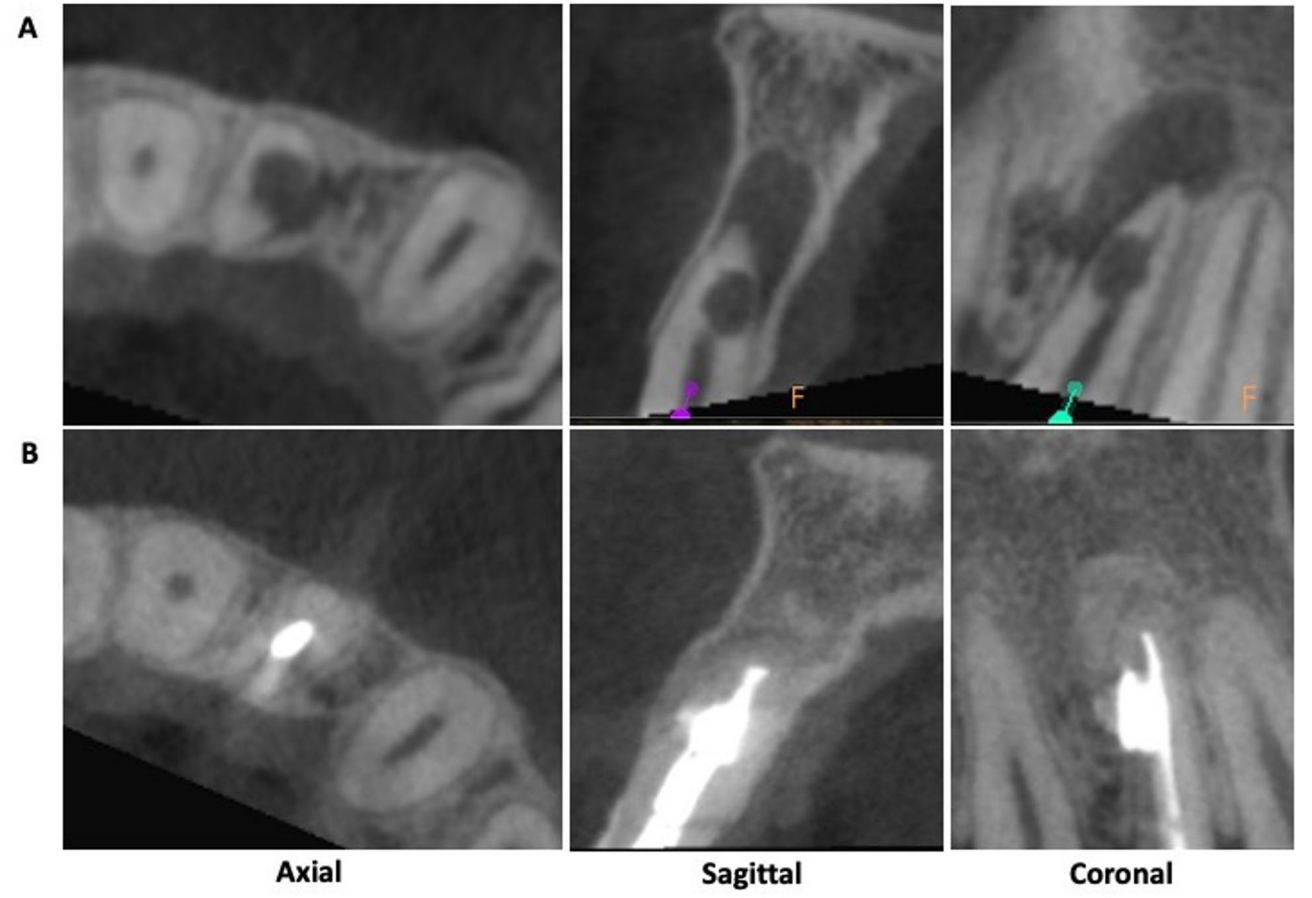
Cone-beam computed tomography (CBCT) evaluation of the perforating internal root resorption lesion and long-term healing outcome. **A** Preoperative CBCT views in axial, sagittal, and coronal planes demonstrating a well-defined resorptive cavity originating from the pulp space and extending toward the external root surface, confirming perforation of the root wall of tooth #7. Three-dimensional imaging allowed precise localization of the defect and assessment of its relationship to the surrounding periodontal structures. A periapical bone defect associated with the same tooth is also evident. **B** CBCT images obtained at the eight-year follow-up in axial, sagittal, and coronal planes demonstrating complete osseous healing, re-establishment of cortical bone continuity, and stable repair of the resorptive defect without evidence of recurrent resorption or periapical pathology

CBCT imaging confirmed the presence of an internal resorptive cavity originating from the pulp space and communicating with the external root surface, consistent with perforating IRR according to the European Society of Endodontology (ESE) classification (2023) (Fig. 2A). The three-dimensional evaluation also facilitated accurate localization of the perforation site and assisted in planning the combined non-surgical and surgical treatment approach.

### Treatment procedures

#### First visit: non-surgical phase

Following informed written consent, local anesthesia was administered using 2% lidocaine with 1:80,000 epinephrine (Xylocaine^®^, Dentsply Sirona, York, PA, USA). Rubber dam isolation was achieved, followed by access cavity preparation under an operating microscope (OPMI PICO, Carl Zeiss Meditec AG, Oberkochen, Germany) at ×10 magnification. Upon entry into the pulp chamber, profuse bleeding from the canal was observed, confirming the presence of inflamed granulation tissue associated with the internal resorptive lesion.

The root canal was negotiated and manually instrumented up to a size 45 stainless-steel K-file (Dentsply Sirona, Ballaigues, Switzerland) (Fig. 1B). Irrigation during the initial visit was performed using 2% chlorhexidine gluconate (Consepsis^®^, Ultradent Products Inc., South Jordan, UT, USA). Chlorhexidine was selected because CBCT findings suggested communication between the resorptive cavity and the periodontal tissues, increasing the risk of irrigant extrusion. The use of sodium hypochlorite was therefore avoided during the initial disinfection phase to minimize the risk of irrigant extrusion into the periradicular tissues through the perforation site.

Following chemomechanical preparation, calcium hydroxide paste (Calasept Plus^®^, Nordiska Dental, Ängelholm, Sweden) was placed as an intracanal medicament to promote disinfection and control of the resorptive process (Fig. 1C). The access cavity was then temporarily sealed using glass ionomer cement (Fuji IX GP^®^, GC Corporation, Tokyo, Japan).

#### Second visit: surgical phase

After two weeks, the patient was asymptomatic; however, persistent exudation from the canal suggested communication between the root canal system and the external root surface. A surgical approach was therefore undertaken to allow direct visualization and repair of the perforating defect.

Under local anesthesia using 2% lidocaine with 1:80,000 epinephrine (Xylocaine^®^, Dentsply Sirona, York, PA, USA), a submarginal full-thickness mucoperiosteal flap was reflected to expose the surgical site. The procedure was performed under an operating microscope (Carl Zeiss Meditec AG, Oberkochen, Germany) to enhance visualization of the perforation and facilitate precise placement of the repair material.

**Fig. 3:**
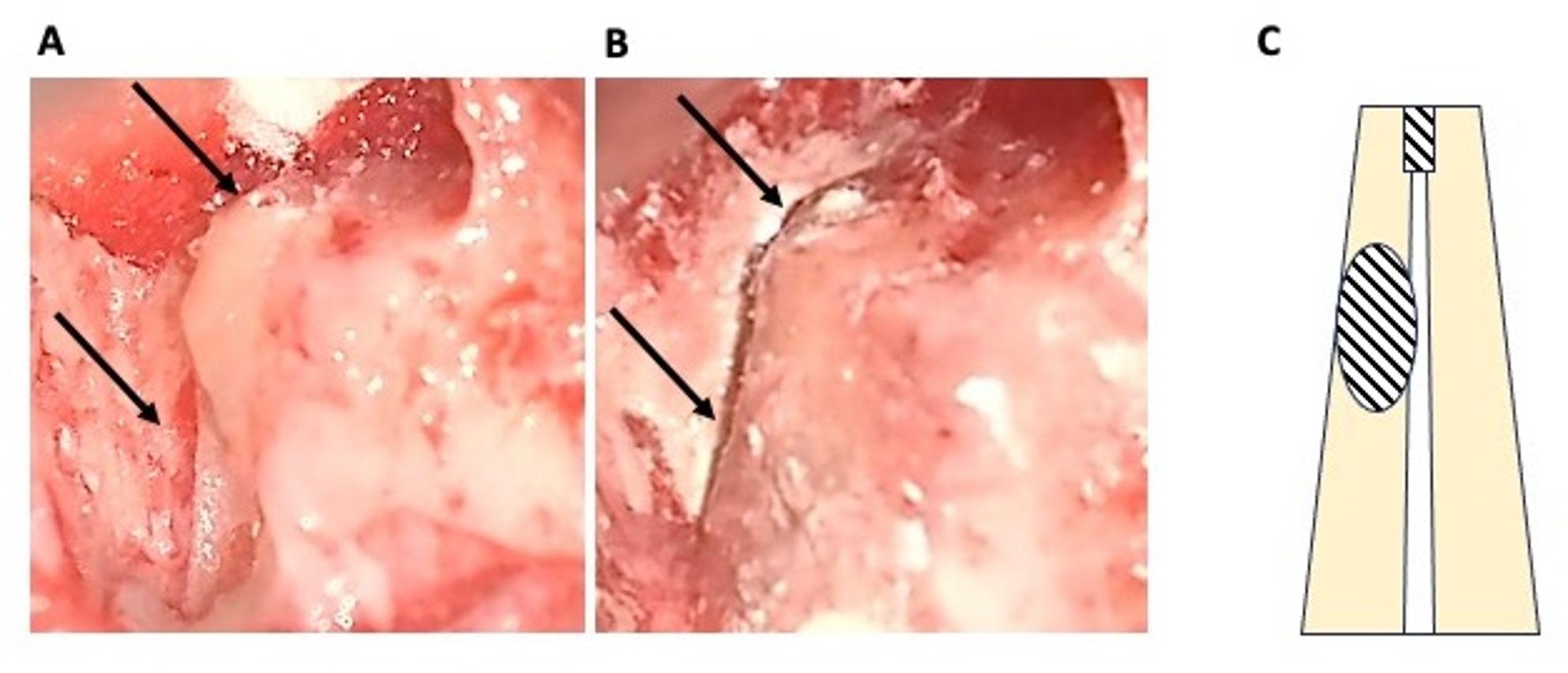
Intraoperative clinical images demonstrating surgical repair of the perforating internal root resorption defect. **A** Surgical exposure of the resorptive defect following flap reflection and removal of inflammatory granulation tissue. The perforation site on the root surface is clearly visible (arrows). **B** Root-end cavity preparation and retrograde filling, followed by sealing of the perforation with EndoSequence Root Repair Material (ERRM) Putty. **C** Schematic illustration depicting the internal resorptive cavity and its communication with the external root surface, together with the surgical repair of the perforation using a bioceramic material

Following flap reflection, inflammatory granulation tissue surrounding the perforation site was carefully removed, allowing clear exposure of the resorptive defect on the external root surface (Fig. 3A). The perforating communication between the root canal system and the periodontal tissues was clearly identified. After thorough debridement of the defect, root-end resection and cavity preparation followed by retrograde filling were also performed to achieve apical sealing of the canal. The perforation was then sealed using a calcium silicate-based bioceramic repair material (EndoSequence Root Repair Material Putty; Brasseler USA, Savannah, GA, USA) (ERRM), placed under magnification to ensure precise adaptation to the defect margins (Fig. 3B). A schematic illustration of the internal resorptive cavity and the surgical repair approach is presented in Fig. 3C.

A xenogeneic bone graft substitute (Geistlich Bio-Oss^®^, Geistlich Pharma AG, Wolhusen, Switzerland) was placed in the osseous defect to provide a scaffold for bone regeneration and to support healing of the periradicular tissues associated with the perforation. The flap was then repositioned and sutured using 4 − 0 chromic gut sutures (Ethicon, Johnson & Johnson, Somerville, NJ, USA). A postoperative periapical radiograph confirmed adequate sealing of the defect (Fig. 1D).

#### Third visit: final obturation

At the one-month follow-up appointment, the patient remained asymptomatic. CBCT evaluation demonstrated satisfactory healing of the periradicular tissues and confirmed stable adaptation of the previously placed repair material.

The root canal was irrigated with 2.5% sodium hypochlorite solution (Clorox^®^, The Clorox Company, Oakland, CA, USA), followed by a final rinse with sterile saline, and subsequently dried with sterile paper points.

Obturation of the root canal system was performed using the hydraulic condensation technique with a calcium silicate-based bioceramic sealer (TotalFill^®^ BC Sealer, FKG Dentaire, La Chaux-de-Fonds, Switzerland) in combination with a single-cone gutta-percha technique (Dentsply Sirona, Ballaigues, Switzerland) (Fig. 1E). The access cavity was then restored using a resin composite restoration (Filtek™ Z350 XT, 3 M ESPE, St. Paul, MN, USA).

### Follow-up

The patient remained clinically asymptomatic at the two-week postoperative review, with no signs of swelling, tenderness to percussion, or sinus tract formation.

At the eight-year follow-up examination, both periapical radiographs and CBCT imaging demonstrated complete resolution of the periapical radiolucency, re-establishment of cortical bone continuity, and restoration of normal trabecular bone architecture surrounding the treated tooth (Figs. 1F and 2B). The tooth remained functional and free of clinical symptoms. Periodontal probing depths remained within normal limits, and no gingival recession or esthetic concerns were observed during the follow-up period.

## Discussion

IRR is an uncommon but clinically significant condition that may compromise tooth structure and long-term prognosis if left untreated [1–4, 6]. The process is initiated by disruption or loss of the odontoblastic layer and predentin, exposing mineralized dentin to odontoclastic activity secondary to pulpal inflammation or trauma [2, 3, 5]. Once perforation occurs, bacterial contamination of the external root surface further complicates the clinical situation, making adequate disinfection and hermetic sealing of the defect essential for long-term success [2, 6]. In the present case, the patient reported a previous history of orthodontic treatment, which has been identified as a potential contributing factor for root resorption. Orthodontic forces may induce pulpal inflammation and stimulate odontoclastic activity, particularly when combined with additional predisposing factors such as previous restorative procedures or trauma [13].

CBCT played a decisive role in the present case by confirming the perforating nature of the resorptive defect and accurately localizing the communication between the root canal system and the periodontal tissues. This three-dimensional assessment directly influenced the treatment strategy and supported the decision to combine orthograde disinfection with surgical repair of the perforation. Previous reports have emphasized that CBCT significantly improves diagnostic accuracy and treatment planning in complex resorptive lesions by enabling precise visualization of defect morphology and its relationship to surrounding periodontal structures [14, 16–18].

The primary therapeutic objective in perforating IRR is to eliminate inflamed tissue, disinfect the root canal system, and hermetically seal the resorptive defect to prevent bacterial ingress [26, 27]. In the present case, initial orthograde treatment using calcium hydroxide as an intracanal medicament helped control inflammation and reduce microbial load, consistent with its well-documented antibacterial properties [28–31]. Persistent exudation during the second visit confirmed external communication, prompting surgical access for direct visualization and repair under the operating microscope. This combined orthograde-surgical approach allowed both internal disinfection and external sealing of the perforation, thereby supporting the structural integrity and biological stability of the tooth. Root-end resection, retrograde cavity preparation, and retrograde filling were also performed during the surgical phase to ensure adequate apical sealing and prevent potential leakage through the apical portion of the canal, thereby contributing to long-term stability of the repair.

Following repair of the perforation, a xenogeneic bone graft was placed in the associated osseous defect to support bone regeneration. Bone graft materials are widely used in regenerative dental procedures because they provide an osteoconductive scaffold that facilitates cellular migration, angiogenesis, and new bone formation within osseous defects [32]. Xenogeneic bone substitutes, such as porcine-derived graft materials, have demonstrated favorable biocompatibility and structural similarity to human bone, allowing them to function as a stable scaffold for bone regeneration. Experimental investigations have shown that porcine bone substitute collagen composites promote osteoconduction and enhance new bone formation both in vitro and in vivo, supporting their use in regenerative procedures involving periapical bone defects [33]. Furthermore, clinical evidence from randomized controlled trials in endodontic microsurgery has suggested that the adjunctive use of bone graft materials may enhance periapical healing and facilitate regeneration of periradicular tissues when used in conjunction with root-end filling materials [34]. In the present case, placement of a xenogeneic bone graft following surgical repair of the perforation may have contributed to stabilization of the osseous defect and supported regeneration of the surrounding periradicular bone, contributing to the favorable long-term healing observed at the eight-year follow-up. The use of bone graft materials in endodontic microsurgery has been suggested to improve osseous defect fill and accelerate periapical bone regeneration, particularly in cases involving large bony defects or cortical plate loss [35].

Mineral trioxide aggregate (MTA) has historically been the material of choice for managing root perforations because of its excellent sealing ability, biocompatibility, and capacity to induce cementogenesis [19, 20]. However, its limitations, including long setting time, potential discoloration, and handling difficulties, have led to the development of next-generation hydraulic calcium silicate-based bioceramic materials, including EndoSequence Root Repair Material (ERRM). ERRM is a premixed, ready-to-use bioactive material composed of calcium silicate, zirconium oxide, and calcium phosphate monobasic, and exhibits high alkalinity and bioactivity [25]. Several clinical and experimental studies have reported comparable or improved handling properties, dimensional stability, and sealing ability for premixed bioceramic repair materials such as ERRM compared with MTA, while maintaining similar biocompatibility and bioactivity [36–38].

The favorable long-term healing observed in the present case may also be related to the bioactive properties of calcium silicate-based materials used for perforation repair. These materials release calcium ions and create an alkaline environment that promotes biomineralization and stimulates hydroxyapatite deposition at the material-tissue interface [39]. Such biological interactions may support cementogenesis and regeneration of surrounding periodontal tissues. Experimental and clinical studies have demonstrated that calcium silicate-based materials can induce mineralized tissue formation and enhance periapical healing when used in root-end repair procedures [40].

Recent reviews have further confirmed the bioactive properties of calcium silicate-based materials, including their ability to release calcium ions, promote biomineralization, and support periapical tissue healing in endodontic repair procedures [40]. These characteristics make such materials particularly suitable for repairing perforating defects and other complex endodontic conditions. In selected cases where perforation is absent or limited, nonsurgical orthograde repair using calcium silicate-based materials may be sufficient. However, in the present case, persistent exudation together with confirmed perforation justified a combined orthograde-surgical approach to enable direct visualization and sealing of the defect. Previous studies have also reported favorable biological and physicochemical properties of calcium silicate-based materials, including satisfactory sealing ability, bioactivity, and promising clinical performance in endodontic applications [23–25]. In addition, a more recent analysis demonstrated that EndoSequence Root Repair Material (ERRM) exhibits stable pH, sustained calcium ion release, and low solubility, supporting its long-term material stability [25]. Experimental studies have further supported the use of calcium silicate-based materials for the management of root perforations, demonstrating favorable bonding characteristics and potential clinical applicability during perforation repair [41]. For instance, Moradi et al., 2026, microscopically evaluated the marginal adaptation of bioceramic sealers, MTA Angelus, and Cold Ceramic in strip perforation repair and reported comparable adaptation among the tested materials, suggesting their suitability for sealing perforation defects [42].

Several case reports have described the management of IRR using contemporary endodontic techniques and bioactive materials. For example, Gehlot et al., 2024, reported successful nonsurgical treatment of a non-perforating internal resorption defect using bioceramic sealer-based obturation with favorable healing after four years of follow-up [43]. Similarly, Asgari and Hajihassani, 2025, described conservative nonsurgical management of perforating IRR associated with dens invagination using a calcium silicate-based material, demonstrating satisfactory healing after 12 months [22]. Other reports have also documented multidisciplinary or surgical approaches for perforating resorptive defects, with follow-up periods of up to three years [44]. In contrast, the present case involved a perforating resorptive lesion requiring a combined orthograde and surgical approach and provides long-term clinical and radiographic evidence of healing over an eight-year follow-up period.

The eight-year clinical and radiographic follow-up in the present case demonstrates that combined non-surgical and surgical management using a bioactive bioceramic repair material resulted in sustained periapical healing and functional preservation of the tooth [23–25]. Such long-term stability is consistent with clinical studies reporting equivalent or superior healing outcomes for EndoSequence Root Repair Material (ERRM) compared with mineral trioxide aggregate (MTA) in endodontic microsurgery [23, 24]. This case also underscores the diagnostic and therapeutic importance of CBCT in identifying perforating IRR, differentiating it from external resorption, and guiding treatment planning [16, 27]. The integration of CBCT imaging, microscopic visualization, and bioactive repair materials may facilitate conservative management of complex perforating lesions while preserving natural tooth structure. In selected cases, perforating IRR may be successfully managed using a combined orthograde and surgical approach when guided by CBCT, enabling long-term tooth preservation and osseous healing [25, 27, 44–46].

Nevertheless, several limitations should be acknowledged. As a single case report, the observed outcome cannot be generalized or attributed exclusively to a specific material or treatment protocol. The favorable healing observed in this case likely reflects the combined effects of accurate diagnosis, effective canal disinfection, appropriate surgical management, and the biological properties of the repair material. Within these limitations, the present case illustrates that perforating internal root resorption can be successfully managed using a combined orthograde and surgical approach supported by modern bioactive bioceramic repair materials, enabling long-term preservation of the affected tooth. Further clinical studies and larger case series are required to confirm the long-term effectiveness of such treatment approaches.

### Clinical implications

This case highlights the diagnostic value of CBCT in identifying perforating IRR and guiding treatment planning. The integration of CBCT imaging, microscopic visualization, and bioactive bioceramic materials may allow conservative management of complex perforating lesions while promoting favorable long-term healing and preservation of the natural tooth.

## Conclusion

The present case illustrates that perforating IRR may be successfully managed using a combined non-surgical and surgical approach guided by cone-beam computed tomography (CBCT) and supported by bioactive bioceramic repair materials such as EndoSequence Root Repair Material (ERRM). The favorable eight-year clinical and radiographic outcome observed in this case suggests that this approach may represent a viable treatment option in selected cases, allowing preservation of the affected tooth and long-term periradicular tissue healing.

## Data Availability

No datasets were generated or analyzed during the current study.
